# Decreased peri-parafoveal RPE, EZ and ELM intensity: A novel predictive biomarker for hydroxychloroquine retinal toxicity

**DOI:** 10.1007/s00417-024-06532-8

**Published:** 2024-06-05

**Authors:** Aysun Yucel Gencoglu, Abdullah Ağın, Dilan Colak, Yasemin Un, Yucel Ozturk

**Affiliations:** 1 Department of Ophthalmology, University of Health Science, Haydarpasa Numune Training and Research Hospital, Istanbul, Turkey; 2 Department of Ophthalmology, University of Health Science, Haseki Training and Research Hospital Fatih, Istanbul, Turkey; 3 Department of Ophthalmology, Medicana Atakoy Hospital, Istanbul, Turkey

**Keywords:** Hydroxychloroquine, Ellipsoid zone, External limiting membrane, Perifoveal, Parafoveal

## Abstract

**Purpose:**

To evaluate the reflectivities of the retinal pigment epithelium (RPE), ellipsoid zone (EZ), and external limiting membrane (ELM) in the central fovea, perifoveal, and parafoveal regions with Optical Coherence Tomography (OCT) and the change in choroid vascular index (CVI) in patients using hydroxychloroquine (HCQ).

**Methods:**

Sixty-one patients underwent HCQ treatment; age and sex-matched 44 control group subjects were included in the study. The RPE, EZ, and ELM reflectivities were measured with the ImageJ program at 5 points, and CVI was calculated.

**Results:**

RPE, EZ, and ELM reflectivities in the central fovea were higher in the HCQ group than in the control group (*p* < 0.001, *p* = 0.013, *p* = 0.022). In the HCQ group, there was a decrease in RPE reflectivities in the temporal, nasal parafovea, and nasal perifovea (*p* = 0.001, *p* = 0.03, *p* = < 0.001). EZ and ELM reflectivity in the nasal parafovea and nasal perifovea was lower in the patient group than in the control group (*p* = 0.007, *p* = 0.005, *p* = 0.009, *p* = 0.001). In the HCQ group, all absolute para and perifoveal reflectivities relative to the fovea decreased significantly more than in the control group (*p* < 0.05).

**Conclusion:**

In patients who underwent HCQ treatment, there is a decrease in the reflectivities of the para and perifoveal RPE, EZ, and ELM compared to the fovea. This decrease is more pronounced than the decrease in reflectivity in the para and perifoveal regions compared to the fovea in people who do not use HCQ. This situation can be considered as a sign of toxicity that is a precursor to overt maculopathy.

## Key Messages

***What is known:***

• Damage to outer retinal structures, such as the ellipsoid zone and external limiting membrane in the parafoveal and perifoveal regions, can occur due to hydroxychloroquine use.

***What is new:***

• In patients who underwent hydroxychloroquine treatment, there is a decrease in the reflectivities of the para and perifoveal retina pigment epithelium, ellipsoid zone, and external limiting membrane compared to the fovea.

• Increased reflectivities of retina pigment epithelium, ellipsoid zone, and external limiting membrane in the fovea may occur due to hydroxychloroquine use. This association, potentially linked to an undisclosed cause, warrants further investigation through studies involving a larger cohort of patients and employing varied methodologies for verification.

• Quantitative evaluation of the hyperreflective bands on optical coherence tomography is a precious clinical instrument, especially for photoreceptor structures in patients undergoing hydroxychloroquine treatment.

## Introduction

Hydroxychloroquine (HCQ) and chloroquine (CQ) have a long history of use in the treatment of autoimmune diseases such as systemic lupus erythematosus, rheumatoid arthritis, and other connective tissue disorders besides being used as antimalarial agents [1, 2]. Although the exact mechanism of action remains unclear, these drugs are known to have immunomodulatory properties [2]. They are being considered for new applications in adjunct cancer therapy and systemic diseases such as diabetes mellitus and cardiovascular disorders [3]. Because CQ has a worse safety profile, HCQ is currently preferred in most treatments. Nevertheless, HCQ has severe side effects.

Retinal toxicity is a significant side effect of HCQ. If retinal toxicity is not detected in the early stages, it can lead to permanent vision loss. Previously, while the diagnosis of retinopathy was based on late-stage Bull’s-eye retinopathy, retinal toxicity was considered to be rare. Recent studies revealed that the prevalence of toxic retinopathy is about 4.3–13.8% [4, 5]. Therefore, many committees have highlighted strict monitoring guidelines to help early diagnosis [3, 4, 6]. A joint committee of major medical societies consisting of rheumatologists, dermatologists, and ophthalmologists suggests following the patients with at least one objective structural test and one subjective functional test [4]. For primary routine screening, spectral domain optical coherence tomography (SD-OCT) and automated 10 − 2 visual field tests are recommended [3].

It is now better understood that higher daily dose usage (> 5 mg/kg actual body weight) increases the risk of retinopathy, besides the duration of use at least 5 years [7]. The drug is excreted from the kidneys, and patients with poor renal function need closer monitoring and lower doses [8]. After detecting toxic retinopathy, cessation of HCQ therapy could prevent vision loss.

Annual screening is recommended to start no more than 5 years after HCQ initiation to detect early changes [3]. Early stages of retinal involvement are subtle, unlike “Bull’s-eye retinopathy.” Thinning of outer retinal layers or mild disruption of the ellipsoid zone (EZ), interdigitation zone (IZ), and photoreceptor layer: These early changes can sometimes be complex and difficult to distinguish from ordinary [9]. Further tests like fundus autofluorescence imaging and multifocal electroretinography should be performed when subtle changes are suspected.

Recent innovations in SD-OCT technology have significantly enhanced our capability to display distinct retinal layers, allowing us to examine the retina more precisely. Choroidal vascularity index (CVI), a novel OCT-based biomarker, is a quantitative analysis of the vascular structure of the choroid [10]. Recent studies revealed that CVI offers promising results in assessing age-related macular degeneration, geographic atrophy follow-ups, and especially ocular inflammatory diseases [1, 11, 12]. CVI is a more accurate biomarker than choroidal thickness because both morphological and vascular changes in choroid are evaluated [10]. Besides, although it is well known that there is a loss of outer retinal structures in the peri and parafoveal regions in HCQ retinopathy, it has not been fully clarified how these structures are affected in cases where apparent retinopathy.

In this study, we analyzed the reflectivities of the retinal pigment epithelium (RPE), EZ, and external limiting membrane (ELM) within the foveal, perifoveal, and parafoveal regions and CVI in patients taking HCQ. We compared them to a control group using ImageJ software. Additionally, we aimed to assess choroidal changes, potentially indicative of early toxic retinopathy, in HCQ users by comparing and quantifying CVI between HCQ patients and healthy controls to investigate potential variations in choroidal conditions during HCQ therapy.

## Materials and methods

### Study Group

The patients’ data was collected retrospectively from the Department of Ophthalmology of Haydarpasa Numune Research and Training Hospital. The principles of the Helsinki Declaration were followed throughout the study. The study was carried out with the approval of the Institutional Ethics Committee (HNEAH-KAEK 2023/KK/101). The authors declare that there is no conflict of interest.

Sixty-one patients underwent HCQ treatment (group 1) for at least 1 year; age and sex-matched 44 control group subjects (group 2) were included in the study. None of the patients using HCQ exhibited any clinical or OCT evidence of toxicity. Participants with a retinal surgery or macular disease history were excluded from the study. None of the participants had other systemic diseases, such as diabetes mellitus or hypertension, and none took systemic medications that could affect the retina. The daily HCQ treatment dose in milligrams per kilogram of body weight (mg/kg), the duration in years, and the cumulative dose in grams were also recorded.

### Data collection and recording

Comprehensive patient records were noted, including best corrected visual acuity (BCVA, Snellen), slit-lamp examination, dilated fundoscopic evaluation, and standard automated perimetry using the 10–2 SITA (Swedish Interactive Threshold Algorithm)-fast program conducted with the Humphrey Visual Field Analyzer 3 (Carl Zeiss Inc., Dublin, CA). The mean deviation (MD) and pattern standard deviation (PSD) values were recorded, along with the number of scotoma points with *p*-values below 1%.

Optical coherence tomography (OCT) scans are obtained with spectral-domain OCT (Spectralis®, Heidelberg Engineering GmbH, Heidelberg, Germany).

### Measurement of ELM, IS/OS, and RPE reflectivity

**Fig. 1:**
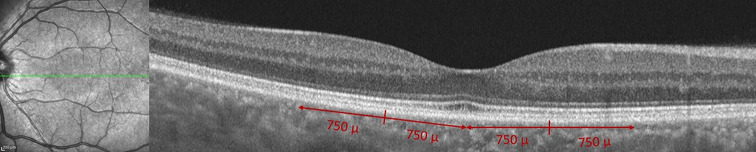
All measurements were made at 5 points at 750-micron intervals from the central fovea on both nasal and temporal sides on the grayscale OCT images. (central fovea, parafovea, perifovea)

Two masked observers (AYG, AA) examined OCT images that were randomly chosen and used the ‘plot profile’ feature in the Image J software (version 1.8.0_77, Bethesda, MD, USA; http://imagej.nih.gov/ij/). To generate a reflectivity graph and measure reflectivity peaks at the ELM, EZ, and RPE along the line, a vertical straight line was drawn, running from the vitreous to the choroid, cutting across the retina. A 750 μm long line was drawn from the central fovea, both nasally and temporally, with a total length of 1500 μm. (Fig. 1) This procedure was performed at three retinal regions of each eye: fovea, perifovea, and parafovea.

**Fig. 2:**
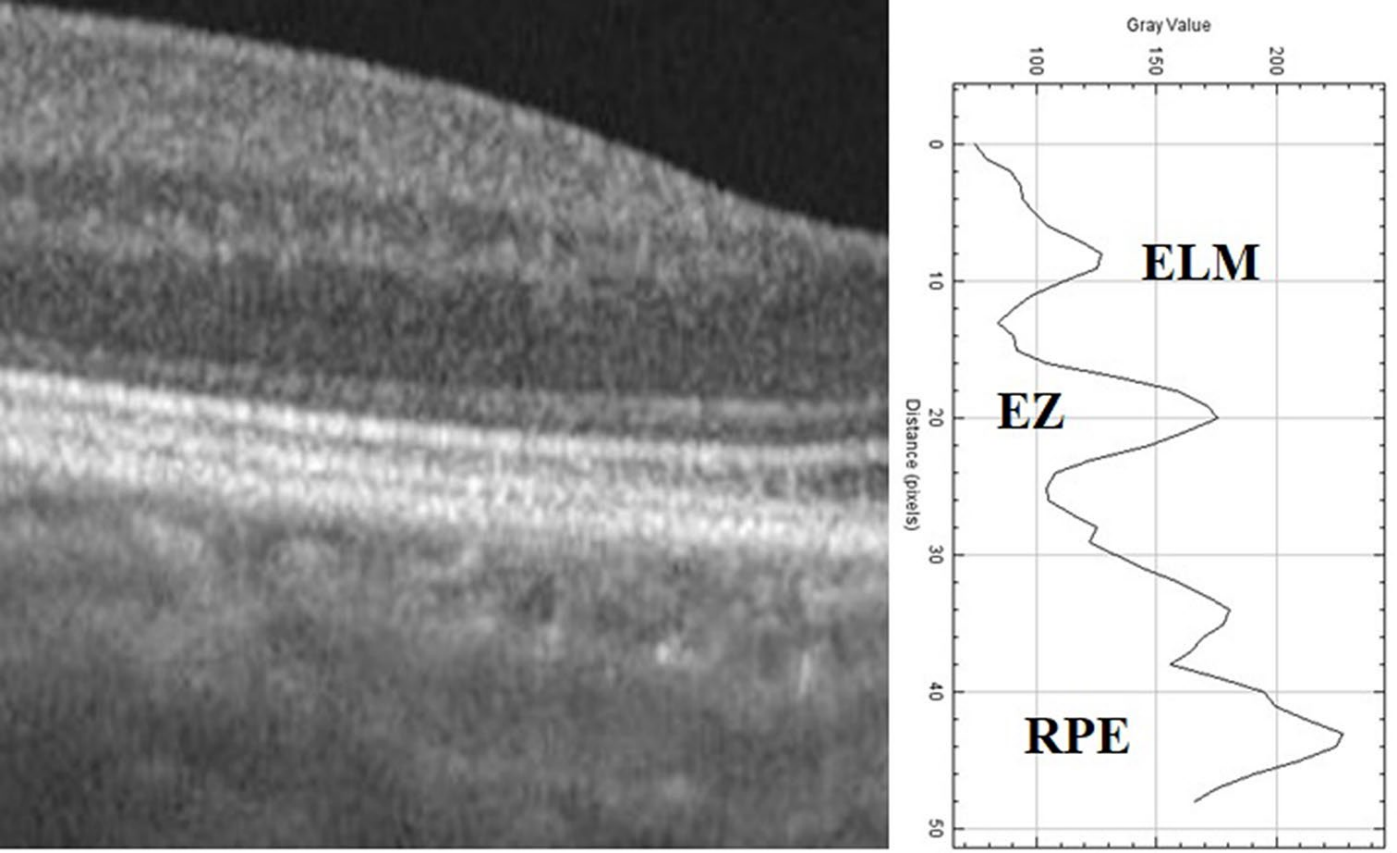
Peaks of the ellipsoid zone (EZ), external limiting membrane (ELM), and retinal pigment epithelium (RPE) on the reflectivity graph (right) and grayscale OCT picture (left) that were produced by an image processing tool (ImageJ). ImageJ generates a reflectivity graph and provides reflectivity values along a line

In a standard OCT image, the histological order of reflectivity is as follows: the RPE layer exhibits the highest reflectivity, followed by the EZ and, subsequently, the ELM. In light of these data, the peak reflectivity on the graph was identified as the highest point corresponding to the RPE layer. Additionally, the precise positions of the RPE layer, EZ, and ELM on the OCT image were concurrently verified by applying the ‘live’ plot profile function within the software, facilitating a comprehensive alignment with the reflectivity graph. (Fig. 2) Relative (Rel) EZ and ELM reflectivity was obtained by dividing the EZ and ELM reflectivities by the RPE reflectivity.

### Measurements of CVI

CVI measurements were calculated using Image J software. The images obtained with EDI-OCT were converted using the Image J software. The area under investigation was determined to have a width of 1500 μm, with 750 μm on the nasal side and 750 μm on the temporal side of the central fovea. The structure extended in a vertical direction from RPE to the border between the choroid and sclera. The choroidal area was defined using the ImageJ ROI Manager.

**Fig. 3:**
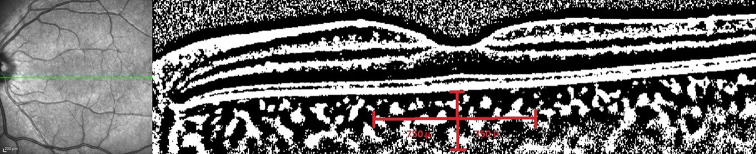
Grayscale OCT picture after binarization by the Image J software. Black markup for the luminal area and white markup for the stromal area was used

Next, three choroidal vessels with lumens above 100 μm were randomly chosen using the Oval Selection Tool on the ImageJ toolbar. The average reflectance of these areas was then calculated. The average brightness was adjusted to its lowest value to reduce the noise in the OCT image. Subsequently, the image underwent an 8-bit conversion and was modified using the Niblack Auto Local Threshold. The binarized image was reconverted to an RGB image consisting of red, green, and blue components. The luminal area was then estimated by using the Threshold Tool. The choroidal luminal and stromal areas were automatically calculated by including the pixel distance data [13]. The CVI value was obtained as the ratio of LA to TCA (Fig. 3).

### Statistical analysis

All analyses were performed using IBM SPSS Statistics 15.0 (Statistical Package for Social Sciences; SPSS Inc IBM, Armonk, NY, USA). Descriptive statistics were stated as mean ± standard deviation (SD) according to the assumption of normal distribution. The normal distribution of variables was assessed using Kolmogorov-Smirnov test. An independent samples t-test was applied to compare mean group values. When the variables were not normally distributed, the Mann–Whitney U test was used to compare differences between two independent groups. Categorical variables were compared using the Chi-square test (Yates continuity correction) or Fisher’s exact test where appropriate. Wilcoxon Test examined the differences between the dependent variables that were not from the normal distribution. Kruskal Wallis test was used to compare more than two independent numerical variables. The average of the measurements made by two independent observers (AYG, AA) was taken in the statistical evaluation. The inter-examiner reproducibility of the measurements was assessed by measuring the intraclass correlation coefficient (ICC). A *p* < 0.05 was accepted as statistically significant.

## Results

### Demographics

Table 1 shows the demographics and characteristics of the participants in the study groups. A total of 105 subjects were included in the study. Sixty-one patients (47 male,14 female) receiving HCQ treatment were included in the study and were compared with 44 age-matched healthy subjects (29 male, 15 female). The ages of the patient group and the male-to-female ratio showed no statistically significant difference compared to the control group (*p* = 0.62). The mean age in the patient group was 57.4 ± 10.8, while in the control group, the mean age was 57.9 ± 11.1 (*p* = 0.71). The average weight was 74.7 ± 19.5 kg, and the average height was 162.2 ± 9.7 cm. The average body mass index was 28.2 ± 6.0, the average duration of drug use was 7.0 ± 4.9 years, and the average cumulative dose was 535.333 ± 442.624 gr/kg in the patient group.

**Table 1:** Demographics and Characteristics of participants in the study groups

	Patient Group		Control Group		
	Mean (± SD)	Range (Min-Max)	Mean (± SD)	Range (Min-Max)	*p*
Age	57.4 ± 10.8	22–79 (59)	57.9 ± 11.1	24–81 (60)	0.71
Gender (Male/Female) n (%)	47 (77) / 14 (23)		29 (65.9) / 15 (34.1)		0.62
MD (Mean Deviation)	-0.72 ± 2.59	-16.29-2.66 (-0.40)			
PSD (Pattern Standard Deviation)	1.50 ± 1.11	0.77–8.78 (1.26)			
Laterality 1/2 n (%)	53 (94.6) / 3 (5.4)				
Reliability n (%)	41 (73.2)				
Scotoma points (*p* < 1%) (numbers)	4.05 ± 7.9	0–49 (1)			
Weight (Kilograms)	74.7 ± 19.5	50–133 (73)			
Height (Centimeters)	162.2 ± 9.7	150–188 (161)			
BMI (Body Mass Index)	28.2 ± 6.0	19.4–40.1 (26.1)			
Duration of Use (Years)	7.0 ± 4.9	1–25 (5)			
Cumulative Dose (gr/kg)	535.333 ± 442.624	0-2190 (365)			
Daily Dose (mg)	209.76 ± 43.61	200–400			

### Reflectivity measurements

The reflectivity measurements of RPE, EZ, and ELM in the central fovea were significantly higher in the patient group compared to the control group (*p* < 0.001, *p* = 0.013, *p* = 0.022, respectively). RPE reflectivity at the temporal and nasal parafovea and the nasal perifovea exhibited significant reductions in the patient group (*p* = 0.001, *p* = 0.03, *p* = < 0.001, respectively). EZ reflectivity at the nasal parafovea and nasal perifovea was notably lower in the patient group (*p* = 0.007, *p* = 0.005, respectively). ELM reflectivity at the nasal parafovea and nasal perifovea showed statistically significant decreases in the patient group (*p* = 0.009, *p* = 0.001, respectively). Relative EZ and ELM reflectivities at central fovea, temporal perifovea, temporal parafovea, nasal perifovea, and nasal parafovea did not show significant difference between patients taking HCQ and control subjects (Table 2). In the correlation analysis, although statistically significant correlations were observed among some parameters, it was not deemed that these correlations could be clinically significant (Table 3). When comparing the reflectivity changes among the central fovea, parafovea, and perifovea regions in the patient group with those in the control group, a statistically significantly more significant decrease in reflectivity was observed in the patient group for all absolute indices, relELM at central fovea vs. relELM at nasal perifovea and relELM at central fovea vs. relELM nasal parafovea (Table 4).

**Table 2:** Comparison of choroidal vascular index and retinal pigment epithelium (RPE), ellipsoid zone (EZ) and external limiting membrane (ELM) reflectivities at central fovea, parafovea and perifovea

	Patient Group		Control Group		
	Mean (± SD)	Range (Min-Max)	Mean (± SD)	Range (Min-Max)	*p*
**CVI**	0.67 ± 0.03	0.62–0.76 (0.67)	0.66 ± 0.02	0.61–0.71 (0.66)	0.057
RPE reflectivity at central fovea (AU)	227.6 ± 11.2	198–251 (226.5)	217.5 ± 12.8	170–243 (219)	< 0.001
EZ reflectivity at central fovea (AU)	186.0 ± 22.1	132–232 (187)	173.9 ± 26.1	111–231 (176.5)	0.013
ELM reflectivity at central fovea (AU)	119.8 ± 16.1	76–150 (121.5)	112.1 ± 17.1	54–141 (115.5)	0.022
RPE reflectivity at temporal parafovea (AU)	213.5 ± 17.5	119–236 (215)	221.9 ± 19.8	118–246 (224)	0.001
EZ reflectivity at temporal parafovea (AU)	198.7 ± 20.5	139–233 (202.5)	203.0 ± 21.5	135–241 (204.5)	0.302
ELM reflectivity at temporal parafovea (AU)	109.8 ± 14.9	69–145 (109.5)	115.2 ± 19.0	48–143 (116.5)	0.113
RPE reflectivity at nasal parafovea (AU)	214.3 ± 12.0	183–238 (213.5)	221.0 ± 9.6	199–243 (221.5)	0.003
EZ reflectivity at nasal parafovea (AU)	190.9 ± 20.5	137–230 (192.5)	202.8 ± 22.8	145–249 (207.5)	0.007
ELM reflectivity at nasal parafovea (AU)	110.6 ± 16.0	65–142 (111.5)	119.7 ± 18.5	69–164 (121.5)	0.009
RPE reflectivity at temporal perifovea (AU)	215.8 ± 12.9	180–239 (216)	220.0 ± 14.4	171–248 (221)	0.123
EZ reflectivity at temporal perifovea (AU)	189.5 ± 22.5	124–236 (189)	198.2 ± 25.0	121–236 (202.5)	0.069
ELM reflectivity at temporal perifovea (AU)	107.6 ± 11.7	72–125 (109.5)	112.5 ± 16.5	52–145 (111.5)	0.081
RPE reflectivity at nasal perifovea (AU)	210.0 ± 14.0	173–235 (210)	221.7 ± 13.3	193–251 (221.5)	< 0.001
EZ reflectivity at nasal perifovea (AU)	187.2 ± 22.5	134–225 (190)	200.1 ± 22.8	129–254 (197.5)	0.005
ELM reflectivity at nasal perifovea (AU)	102.9 ± 15.5	62–144 (102.5)	114.1 ± 16.4	66–165 (115.5)	0.001
Relative EZ reflectivity at central fovea (%)	81.9 ± 10.1	55.2-102.7 (83.2)	79.9 ± 10.9	54.1-110.5 (81.4)	0.350
Relative ELM reflectivity at central fovea (%)	52.6 ± 6.7	35.2–66.1 (51.6)	51.5 ± 7.1	31.8–62.9 (52.0)	0.396
Relative EZ reflectivity at temporal perifovea (%)	87.9 ± 9.6	55.1-109.3 (88.3)	90.3 ± 11.2	52.4-107.9 (91.7)	0.158
Relative EZ reflectivity at temporal parafovea (%)	93.9 ± 15.8	72.0-195.8 (92.0)	92.8 ± 18.5	59.0-191.5 (91.3)	0.482
Relative ELM reflectivity at temporal perifovea (%)	49.9 ± 5.0	38.7–59.8 (50.6)	51.1 ± 6.6	30.4–64.4 (50.2)	0.306
Relative ELM reflectivity at temporal parafovea (%)	51.6 ± 6.9	35.2–75.6 (50.4)	52.3 ± 10.7	27.3–98.3 (53.6)	0.681
Relative EZ reflectivity at nasal perifovea (%)	89.4 ± 10.8	59.6-111.5 (90.7)	90.6 ± 12.1	61.1-119.8 (90.6)	0.576
Relative EZ reflectivity at nasal parafovea (%)	89.2 ± 9.2	67.8-109.6 (89.3)	91.8 ± 10.3	67.3-110.2 (93.5)	0.174
Relative ELM reflectivity at nasal perifovea (%)	49.0 ± 7.1	33.5–72.0 (49.5)	51.7 ± 8.4	33.3–80.1 (51.8)	0.087
Relative ELM reflectivity at nasal parafovea (%)	51.6 ± 7.1	35.5–65.3 (51.4)	54.1 ± 7.9	34.273.9 (54.7)	0.095

*CVI* choroidal vascular index, *ELM* external limiting membrane, *EZ* ellipsoid zone, *RPE* retina pigment epithelium, *Para* parafoveal, *Peri* perifoveal, *N* nasal, *T* temporal, *Rel* relative, *SD* standard deviation, *AU* arbitary unit

**Table 3:** Relationship of parameters in the patient group (Spearman’s correlation)

			Age	MD	PSD	Scotoma	BMI	Dose	Duration (year)	Dose
CVI		r	-0.002	-0.049	-0.043	-0.108	0.195	0.178	-0.116	-0.120
		**p**	**0.987**	**0.724**	**0.756**	**0.435**	**0.410**	**0.265**	**0.431**	**0.448**
RPE central fovea		r	-0.045	0.187	-0.146	-0.234	0.532	-0.105	-0.070	0.033
		**p**	**0.735**	**0.176**	**0.291**	**0.088**	**0.016**	**0.512**	**0.638**	**0.837**
EZ central fovea		r	0.173	0.204	-0.005	-0.152	-0.205	-0.010	0.119	0.087
		**p**	**0.193**	**0.139**	**0.973**	**0.274**	**0.387**	**0.953**	**0.419**	**0.582**
ELM central fovea		r	0.046	0.083	-0.013	-0.052	0.322	0.153	-0.075	-0.004
		**p**	**0.730**	**0.551**	**0.924**	**0.711**	**0.167**	**0.339**	**0.613**	**0.980**
RPE para T		r	-0.005	0.218	-0.102	-0.179	0.131	0.005	-0.206	-0.154
		**p**	**0.969**	**0.113**	**0.465**	**0.196**	**0.582**	**0.976**	**0.159**	**0.330**
EZ para T		r	0.388	0.165	-0.109	-0.171	0.092	-0.081	-0.164	-0.073
		**p**	**0.003**	**0.234**	**0.435**	**0.217**	**0.700**	**0.613**	**0.265**	**0.646**
ELM para T		r	0.057	0.143	-0.029	-0.189	0.189	-0.072	-0.137	-0.072
		**p**	**0.670**	**0.302**	**0.836**	**0.170**	**0.425**	**0.655**	**0.352**	**0.650**
RPE para N		r	-0.053	-0.137	0.221	0.125	0.331	-0.235	0.130	0.130
		**p**	**0.693**	**0.322**	**0.109**	**0.368**	**0.153**	**0.140**	**0.377**	**0.412**
EZ para N		r	0.292	0.220	-0.159	-0.215	-0.063	-0.029	-0.206	-0.085
		**p**	**0.026**	**0.110**	**0.251**	**0.119**	**0.791**	**0.859**	**0.160**	**0.592**
ELM para N		r	0.061	0.220	-0.053	-0.081	0.276	0.139	-0.010	0.049
		**p**	**0.649**	**0.110**	**0.705**	**0.561**	**0.238**	**0.387**	**0.949**	**0.758**
RPE peri T		r	0.190	0.134	0.080	0.005	0.154	-0.101	-0.204	-0.187
		**p**	**0.153**	**0.333**	**0.567**	**0.970**	**0.518**	**0.532**	**0.165**	**0.235**
EZ peri T		r	0.230	0.366	-0.231	-0.339	0.178	-0.067	-0.126	-0.131
		**p**	**0.083**	**0.006**	**0.093**	**0.012**	**0.454**	**0.677**	**0.393**	**0.410**
ELM peri T		r	0.150	0.001	0.086	0.017	0.004	-0.168	0.050	0.114
		**p**	**0.261**	**0.993**	**0.537**	**0.902**	**0.987**	**0.295**	**0.734**	**0.473**
RPE peri N		r	0.060	0.001	-0.047	-0.003	0.326	0.173	0.151	0.193
		**p**	**0.657**	**0.995**	**0.734**	**0.980**	**0.160**	**0.281**	**0.307**	**0.220**
EZ peri N		r	0.250	0.198	-0.332	-0.317	0.144	-0.096	0.010	-0.033
		**p**	**0.059**	**0.151**	**0.014**	**0.020**	**0.544**	**0.552**	**0.944**	**0.837**
ELM peri N		r	0.151	0.080	-0.025	-0.116	0.463	0.230	0.091	0.148
		**p**	**0.256**	**0.566**	**0.858**	**0.405**	**0.040**	**0.148**	**0.539**	**0.349**
relEZ central fovea		r	0.167	0.081	0.049	-0.045	-0.447	0.019	0.115	0.053
		**p**	**0.210**	**0.561**	**0.727**	**0.746**	**0.048**	**0.905**	**0.435**	**0.741**
relELM central fovea		r	0.074	0.060	0.019	-0.014	0.244	0.172	-0.084	-0.024
		**p**	**0.579**	**0.668**	**0.891**	**0.919**	**0.301**	**0.282**	**0.569**	**0.880**
relEZperi T		r	0.213	0.289	-0.219	-0.306	-0.131	-0.029	-0.054	-0.052
		**p**	**0.109**	**0.034**	**0.111**	**0.025**	**0.582**	**0.859**	**0.716**	**0.744**
relEZpara T		r	0.496	0.097	-0.097	-0.108	0.114	-0.182	-0.043	0.001
		**p**	**< 0.001**	**0.486**	**0.486**	**0.436**	**0.631**	**0.255**	**0.773**	**0.995**
relELMperiT		r	0.047	0.026	-0.068	-0.099	-0.227	-0.048	0.175	0.264
		**p**	**0.724**	**0.854**	**0.623**	**0.478**	**0.336**	**0.766**	**0.235**	**0.091**
relELMparaT		r	0.081	0.057	-0.009	-0.132	0.182	0.010	-0.106	-0.040
		**p**	**0.544**	**0.683**	**0.946**	**0.341**	**0.443**	**0.953**	**0.474**	**0.801**
relEZperi N		r	0.226	0.253	-0.316	-0.308	0.111	-0.086	-0.098	-0.164
		**p**	**0.088**	**0.065**	**0.020**	**0.023**	**0.640**	**0.592**	**0.506**	**0.301**
relEZpara N		r	0.344	0.342	-0.333	-0.336	-0.253	0.000	-0.300	-0.193
		**p**	**0.008**	**0.011**	**0.014**	**0.013**	**0.283**	**1.000**	**0.038**	**0.220**
relELMperi N		r	0.161	0.087	0.009	-0.103	0.411	0.211	0.036	0.061
		**p**	**0.228**	**0.533**	**0.949**	**0.458**	**0.071**	**0.186**	**0.808**	**0.699**
relELMpara N		r	0.065	0.276	-0.164	-0.146	0.144	0.239	-0.092	-0.014
		**p**	**0.629**	**0.044**	**0.237**	**0.291**	**0.544**	**0.132**	**0.535**	**0.928**

*CVI* choroidal vascular index, *ELM* external limiting membrane, *EZ* ellipsoid zone, *RPE* retina pigment epithelium, *Para* parafoveal, *Peri* perifoveal, *N* nasal, *T* temporal, *Rel* relative, *SD* standard deviation

**Table 4:** Reflectivity differences: central fovea, parafovea, and perifovea - control vs. patient groups

	Patient Group Difference					Control Group Difference					
	Mean	SD	95% CI		*p*	Mean	SD	95% CI		*p*	Patient vs. Control *p*
EZc vs. EZ para T	-12.7	25.9	-19.5	-5.9	< 0.001	-29.2	26.5	-37.2	-21.1	< 0.001	0.002
EZc vs. EZ para N	-4.9	28.5	-12.4	2.6	0.198	-28.9	30.2	-38	-19.7	< 0.001	< 0.001
EZc vs. EZ peri T	-3.6	28.3	-11	3.9	0.344	-24.3	29.7	-33.4	-15.3	< 0.001	0.001
EZc vs. EZ peri N	-1.3	28.4	-8.7	6.2	0.737	-26.2	28.3	-34.8	-17.6	< 0.001	< 0.001
ELMc vs. ELM para T	10	14.5	6.2	13.8	< 0.001	-3.1	14.4	-7.4	1.3	0.165	< 0.001
ELMc vs. ELM para N	9.2	15.4	5.2	13.3	< 0.001	-7.6	17.5	-12.9	-2.3	0.006	< 0.001
ELMc vs. ELM peri T	12.2	19	7.2	17.2	< 0.001	-0.4	16.7	-5.5	4.7	0.865	0.001
ELMc vs. ELM peri N	16.9	15.6	12.8	21	< 0.001	-2	15.9	-6.9	2.8	0.404	< 0.001
RPEc vs. RPE para T	14.1	20.2	8.8	19.4	< 0.001	-4.5	20.5	-10.7	1.8	0.002	< 0.001
RPEc vs. RPE para N	13.3	14.2	9.6	17.1	< 0.001	-3.5	14.2	-7.9	0.8	0.106	< 0.001
RPEc vs. RPE peri T	11.8	16.1	7.6	16	< 0.001	-2.5	15.2	-7.2	2.1	0.273	< 0.001
RPEc vs. RPE peri N	17.6	15.2	13.6	21.6	< 0.001	-4.2	15.1	-8.8	0.4	0.070	< 0.001
relEZc vs. relEZperi T	-6.1	13.3	-9.6	-2.6	0.001	-10.4	14	-14.6	-6.1	< 0.001	0.116
relEZc vs. relEZpara T	-12	19.5	-17.1	-6.9	< 0.001	-12.9	19.7	-18.9	-6.9	< 0.001	0.805
relEZc vs. relEZperi N	-7.5	13.1	-10.9	-4.1	< 0.001	-10.7	13.1	-14.7	-6.7	< 0.001	0.265
relEZc vs. relEZpara N	-7.3	13.2	-10.8	-3.8	< 0.001	-11.9	12.1	-15.6	-8.2	< 0.001	0.073
relELMc vs. relELMperi T	2.7	8.2	0.6	4.9	0.014	0.4	7.5	-1.9	2.7	0.734	0.139
relELMc vs. relELMpara T	1	8.8	-1.3	3.3	0.38	-0.9	10.6	-4.1	2.3	0.587	0.326
relELMc vs. relELMperi N	3.6	7.2	1.7	5.5	< 0.001	-0.2	8	-2.7	2.2	0.853	0.013
relELMc vs. relELMpara N	1	7.1	-0.8	2.9	0.142	-2.7	7.6	-5	-0.3	0.806	0.028

*ELM* external limiting membrane, *EZ* ellipsoid zone, *RPE* retina pigment epithelium, *Para* parafoveal, *Peri* perifoveal, *C* central, *N* nasal, *T* temporal, *Rel* relative, *SD* standard deviation

### CVI measurements

CVI was higher in the HCQ group than in the control group; however, this difference was not statistically significant. (*p* = 0.057) (Table 2).

Inter-examiner ICC was greater for each measured parameter than 0.90 (95% confidence interval 0.90–0.92).

## Discussion

This study evaluated reflectivity measurements of the RPE, EZ, and ELM within the central fovea, perifoveal, and parafoveal regions. Subsequently, we compared these measurements to those obtained from a healthy control group. Our analysis demonstrates higher reductions in RPE, ELM, and EZ reflectivity among parafovea and perifovea regions in patients taking HCQ compared to the control group. These findings provide valuable information about the retinal structural changes associated with HCQ use and underscore the importance of monitoring these changes to ensure patients’ safety.

While the exact mechanisms underlying the development of HCQ retinopathy are not fully understood, it is established that the principal site of drug-induced toxicity occurs within the photoreceptor layers [3]. SD-OCT imaging of the macula is recommended as a screening test for patients undergoing systemic CQ or HCQ treatment [3]. The HCQ toxicity has been documented in numerous prior studies using SD-OCT. These studies have highlighted OCT findings such as the absence of the ELM, disruption of the parafoveal EZ, parafoveal attenuation of the outer nuclear layer (ONL), and RPE damage [14–16].

Lally et al. [14] monitored thirty individuals with HCQ retinopathy with SD-OCT after discontinuing the medication. Prior to the disruption of the parafoveal EZ, observations included thinning of ONL, disruption of the parafoveal IZ, and decreased reflectivity of the parafoveal EZ. This deterioration included damage to the parafoveal EZ and retinal pigment epithelium, as well as thinning of ONL. Eyes exhibiting clear signs of toxicity experienced more pronounced thinning in the outer ring of the inferior region 12 months after discontinuing the drug, in comparison to cases of early toxicity (*p* = 0.002, 95% CI − 2 to − 8 μm). The nasal inner subfield exhibited more thinning than the temporal inner subfield at 12 months following discontinuation of the drug, indicating apparent toxicity (*p* = 0.018, 95% CI − 1 to − 8 μm). Garrity et al. [15] evaluated 17 eyes of 10 patients using HCQ. Upon initial detection of SD-OCT anomalies, 82% of the eyes exhibited attenuation of the parafoveal EZ, while 100% of the eyes showed loss of the parafoveal IZ. Moreover, Pham et al. [16] demonstrated that after discontinuing HCQ, early and moderate instances showed stability in fundus autofluorescence appearance, foveal thickness, EZ line length, and visual acuity for a period of up to 9 years. In contrast, severe instances exhibited a persistent decline in these indicators for a duration of up to 20 years after discontinuing the medication. Initial examination revealed that the presence of RPE damage was a strong indicator of progressive retinopathy over an extended period.

Modi et al. [17] found that HCQ toxicity leads to outer and inner retinal volumetric thinning compared to age-matched control patients and patients taking HCQ without exhibiting toxicity. Moreover, Borelli et al. [18] reported that in HCQ patients, there was a significant decrease in the ONL in the foveal (*p* = 0.008), parafoveal (*p* < 0.0001), and perifoveal (*p* < 0.0001) regions. The HCQ cohort was further split into two categories in this investigation based on structurally identifiable retinopathy (i.e., structural damage as detected by multimodal imaging). In the foveal (*p* = 0.032), parafoveal (*p* < 0.0001), and perifoveal (*p* < 0.0001) regions, the ONL thickness was lower in HCQ eyes without retinopathy. The R2:R5 ring ratio of mfERG P1 amplitude was associated with INL (*p* = 0.002) and ONL (*p* = 0.044) thicknesses, while the R3:R5 ring ratio of P1 amplitude was associated with ONL thickness (*p* = 0.004). These structural alterations were significantly associated with macular function in HCQ patients without retinopathy. Besides, in the study, an isolated evaluation of the outer retinal structures was conducted with the specific aim of analyzing the changes quantitatively and comprehensively within these structures.

The reflectivities of RPE, ELM, and EZ were significantly higher in the group using HCQ compared to the control group. This finding constitutes the most intriguing and challenging aspect of the study to interpret. Primarily, this situation may be associated with individual and genetic factors among participants in the patient and control groups. Although somewhat complex to discern, it is also confirmed by these results that the change in the temporal and nasal quadrants relative to the central quadrant is greater in the patient group compared to the control group (Central reflectivities are higher in the patient group compared to the control group, whereas nasal and temporal reflectivities are lower, indicating a higher gap between quadrants in the patient group). Besides, clinically, RPE damage delineating progressive disease remains unclear whether it arises directly from HCQ toxicity to RPE cells or secondarily from adjacent photoreceptor death; however, perhaps due to an as yet unidentified cause, such the scenario could manifest in this context.

As previously stated, the analysis in Table 4 examined the difference in reflectivity changes between the nasal and temporal quadrants relative to the central fovea in both the patient and control groups, demonstrating distinct variations in both groups. It is acknowledged that the values of the control group were considered as a baseline, and it is already known and expected that there would be differences in reflectivities in the temporal and nasal quadrants due to cone and rod densities relative to the central fovea in normal individual. We evaluated whether the expected differences in Table 4 were similar to those in normal individuals or differed. The findings supported the conclusion mentioned above by demonstrating a greater gap in reflectivity in the patient group. However, since one of the changes in the comparisons of relELMc vs. relELMperi N and relELMc vs. relELMpara N in either the patient or/and control group was not significant, the difference in changes between groups, despite being statistically significant, may not be clinically significant. However, the explanations for the evaluated points are provided as legends in the tables. Also, relative indices provide the ratio of EZ and ELM to RPE, indicating how these changes vary across layers. The lack of difference in relative indices suggests that the change in reflectivity across quadrants occurred at a similar rate between groups.

The functions of the RPE, EZ, and ELM, which are the hyperreflective bands of the outer retina, can be evaluated indirectly by measuring the intensities of these structures, like in our study [19]. Hood et al. [20] showed that decreased cone function was correlated with the intensity of the inner segment ellipsoid (ISe) band. With this, Yılmaz Tugan et al. [21] and Toprak et al. [22] determined that the reflectivities of the EZ (formerly known as IS/OS) may be related to prognosis in patients with macular hole and epiretinal membrane. For these reasons, the reflectivities of these structures reflect their functions.

In the current literature, studies have identified the thinning of the outer retinal layers and disruption of the parafoveal EZ and RPE, which have been considered irreversible [3]. It can be valuable to identify subclinical deteriorations, even when OCT appears normal. None of the patients in our study had any clinical findings or signs of toxicity visible in OCT scans. However, in agreement with the literature, our study identified reduced intensity in the ELM, EZ, and RPE in both parafoveal and perifoveal regions in the HCQ treatment group. Besides, no clinically significant correlations were found, particularly between cumulative dose reflectivity decreases. This statement suggests that the etiopathogenesis of the disease may occur depending on many factors, such as genetics and environmental conditions.

We analyzed CVI in HCQ patients and compared the results with those of the control group. Our study showed statistically similar CVI in individuals using HCQ compared to the control group. Recently, Halouani et al. [1] discovered that the CVI was significantly lower in the advanced stage of the HCQ toxicity group. They suggested that CVI could be the potential diagnostic marker of severity because choroidal impairment was shown, especially in the advanced stages of toxic retinopathy. Besides, Hasan et al. [23] showed that choroidal volume and vascularity index were significantly reduced in patients on HCQ therapy, especially at higher cumulative doses. On the other hand, there were no apparent signs of retinopathy in our HCQ group. Thus, it is thought that retinal findings may be more leading changes than choroidal findings.

HCQ toxicity is still a serious concern due to the lack of treatment. However, there is some evidence that central vision can be preserved if the damage can be detected before RPE alterations [3]. Hence, the early detection of toxicity is crucially important. Considering the findings of our study, intensity analysis may provide valuable quantitative data for screening patients undergone HCQ treatment. No data demonstrates the quantitative evaluation of the behavior of outer retinal structures in patients who underwent HCQ therapy; our study is the first in this regard.

## Conclusion

The limitations of this study include its retrospective, single-center design and the relatively small sample size.

In summary, intensity analysis detects decreased ELM, EZ, and RPE reflectivity in parafovea and perifovea in patients taking HCQ. Considering the quantitative nature of this technology, it has the potential to yield objective findings in HCQ screening for the early detection of disease. Further, longitudinal studies are necessary to provide additional validation for the efficacy of this technology.

## Abbreviations

CVI: Choroidal vascular index
ELM: External limiting membrane
EZ: Ellipsoid zone
RPE: Retina pigment epithelium
Para: Parafoveal
Peri: Perifoveal
N: Nasal
T: Temporal
Rel: Relative
SD: Standard deviation
